# Epigenetic regulation of 5α reductase-1 underlies adaptive plasticity of reproductive function and pubertal timing

**DOI:** 10.1186/s12915-021-01219-6

**Published:** 2022-01-07

**Authors:** Ben Bar-Sadeh, Or E. Amichai, Lilach Pnueli, Khurshida Begum, Gregory Leeman, Richard D. Emes, Reinhard Stöger, Gillian R. Bentley, Philippa Melamed

**Affiliations:** 1 Faculty of Biology, Technion-Israel Institute of Technology, 32000 Haifa, Israel; 2 Department of Anthropology, Durham University, Durham, DH1 3LE UK; 3 School of Biosciences, University of Nottingham, Nottingham, LE12 5RD UK; 4 School of Veterinary Medicine and Sciences, University of Nottingham, Nottingham, LE12 5RD UK

**Keywords:** Reproduction, Mice, Colitis, Women, Puberty, Menopause, Ovary, Hypothalamus, Aging, 5α reductase-1

## Abstract

**Background:**

Women facing increased energetic demands in childhood commonly have altered adult ovarian activity and shorter reproductive lifespan, possibly comprising a strategy to optimize reproductive success. Here, we sought to understand the mechanisms of early-life programming of reproductive function, by integrating analysis of reproductive tissues in an appropriate mouse model with methylation analysis of proxy tissue DNA in a well-characterized population of Bangladeshi migrants in the UK. Bangladeshi women whose childhood was in Bangladesh were found to have later pubertal onset and lower age-matched ovarian reserve than Bangladeshi women who grew-up in England. Subsequently, we aimed to explore the potential relevance to the altered reproductive phenotype of one of the genes that emerged from the screens.

**Results:**

Of the genes associated with differential methylation in the Bangladeshi women whose childhood was in Bangladesh as compared to Bangladeshi women who grew up in the UK, 13 correlated with altered expression of the orthologous gene in the mouse model ovaries. These mice had delayed pubertal onset and a smaller ovarian reserve compared to controls. The most relevant of these genes for reproductive function appeared to be *SRD5A1,* which encodes the steroidogenic enzyme 5α reductase-1. *SRD5A1* was more methylated at the same transcriptional enhancer in mice ovaries as in the women’s buccal DNA, and its expression was lower in the hypothalamus of the mice as well, suggesting a possible role in the central control of reproduction. The expression of *Kiss1* and *Gnrh* was also lower in these mice compared to controls, and inhibition of 5α reductase-1 reduced *Kiss1* and *Gnrh* mRNA levels and blocked GnRH release in GnRH neuronal cell cultures. Crucially, we show that inhibition of this enzyme in female mice *in vivo* delayed pubertal onset.

**Conclusions:**

SRD5A1/5α reductase-1 responds epigenetically to the environment and its downregulation appears to alter the reproductive phenotype. These findings help to explain diversity in reproductive characteristics and how they are shaped by early-life environment and reveal novel pathways that might be targeted to mitigate health issues caused by life-history trade-offs.

**Supplementary Information:**

The online version contains supplementary material available at 10.1186/s12915-021-01219-6.

## Background

Phenotypic plasticity allows short- or long-term adaptations in response to perturbations and energetic challenges that often involve life-history trade-offs [1–4]. Adult reproductive function is shaped during early-life in response to such signals, and this can result in changes in timing of sexual maturation, hormone levels, rates of ovulation and fertility, and length of the reproductive lifespan [4–7]. The effect of the early-life environment on adult developmental programming, particularly at mid-childhood, has been reported [8, 9] and was evident in a series of studies on Bangladeshi migrants whose reproductive phenotype was associated with whether they had grown up in Bangladesh or the UK [10, 11]. Bangladeshi women who grew up in Bangladesh experienced later pubertal onset and earlier menopause and had a lower age-matched ovarian reserve than those who grew up in the UK, while women who migrated as adults retained this phenotype even after many years in the UK [10, 12, 13]. Bangladesh is prone to seasonal floods and has a diverse pathogenic environment [14, 15] with many outbreaks of disease and relatively poor healthcare [16]. This reproductive phenotype was associated with higher recalled infectious and parasitic disease loads during childhood in Bangladesh [10, 12, 13] suggesting that increased energetic demands due to frequent immune responses might be responsible for trade-offs leading to altered investment in reproduction [17, 18].

Developmental programming, especially that affected by environmental signals, indicates a likely role for the epigenome which is modified during normal reproductive development and maturation [19–23]. The epigenome is highly sensitive to the metabolic state and cellular environment [24–26] and so is positioned to mediate adaptive responses in accordance with changes in local ecologies [5, 27]. We thus hypothesized that epigenetic programming might underlie this reproductive phenotypic plasticity and allow strategic programming in accordance with signals from the early-life environment.

Given the inherent problems of reproductive tissue inaccessibility in healthy human subjects, we set out to identify genes associated with altered DNA methylation in buccal tissue of Bangladeshi migrant women who had grown up in Bangladesh or the UK and to compare and integrate these findings with changes in gene expression in the reproductive tissues of a suitable mouse model. We first established such a mouse model based on findings that the “Bangladeshi childhood” reproductive phenotype, including delayed menarche and reduced ovarian reserve, was associated with increased inflammatory burden from infectious and parasitic diseases prior to puberty [10, 12, 13] and did not appear to be due to differential exposure to endocrine disruptors [28, 29]. We therefore adopted a mouse model of temporary colitis in newly weaned female mice by administration of dextran sulfate sodium (DSS). Given that DSS-treatment induces an inflammatory response mimicking gastrointestinal infection, this was a suitable model for the aims of our study, particularly as DSS-induced colitis was reported previously to delay pubertal onset in mice [30].

Our first aim was to examine whether genes associated with differential methylation in the Bangladeshi women’s proxy tissue DNA showed accordant changes in expression in the mouse reproductive tissues which could indicate epigenetically regulated genes that might underlie the commonly altered phenotype. We could then pursue our second aim which was to test causality in the mouse model and elucidate the mechanisms involved.

In this way, we discovered that expression of *SRD5A1* was downregulated in the ovaries of the mouse model, correlating with an increase in its methylation at the same *locus* as in the DNA of Bangladeshi women who had grown up in Bangladesh. This gene encodes 5α reductase-1, a steroidogenic enzyme that catalyzes production of steroids that repress ovarian follicle recruitment [31, 32] and of brain neurosteroids [33], so we hypothesized that its epigenetic regulation might underlie the altered reproductive characteristics. We found that *SRD5A1* was also downregulated in the region of the hypothalamus that controls the reproductive axis and demonstrated in cell lines its effects on gonadotropin-releasing hormone (GnRH) and Kiss1 expression. Subsequently, we showed that inhibition of 5α reductase-1 delays pubertal onset. The environmental sensitivity of *SRD5A1* demonstrates how pubertal timing and adult ovarian function might be programmed, to determine the duration of the reproductive lifespan.

## Results

### Pre-pubertal colitis in mice affects pubertal timing and ovarian function

**Fig. 1:**
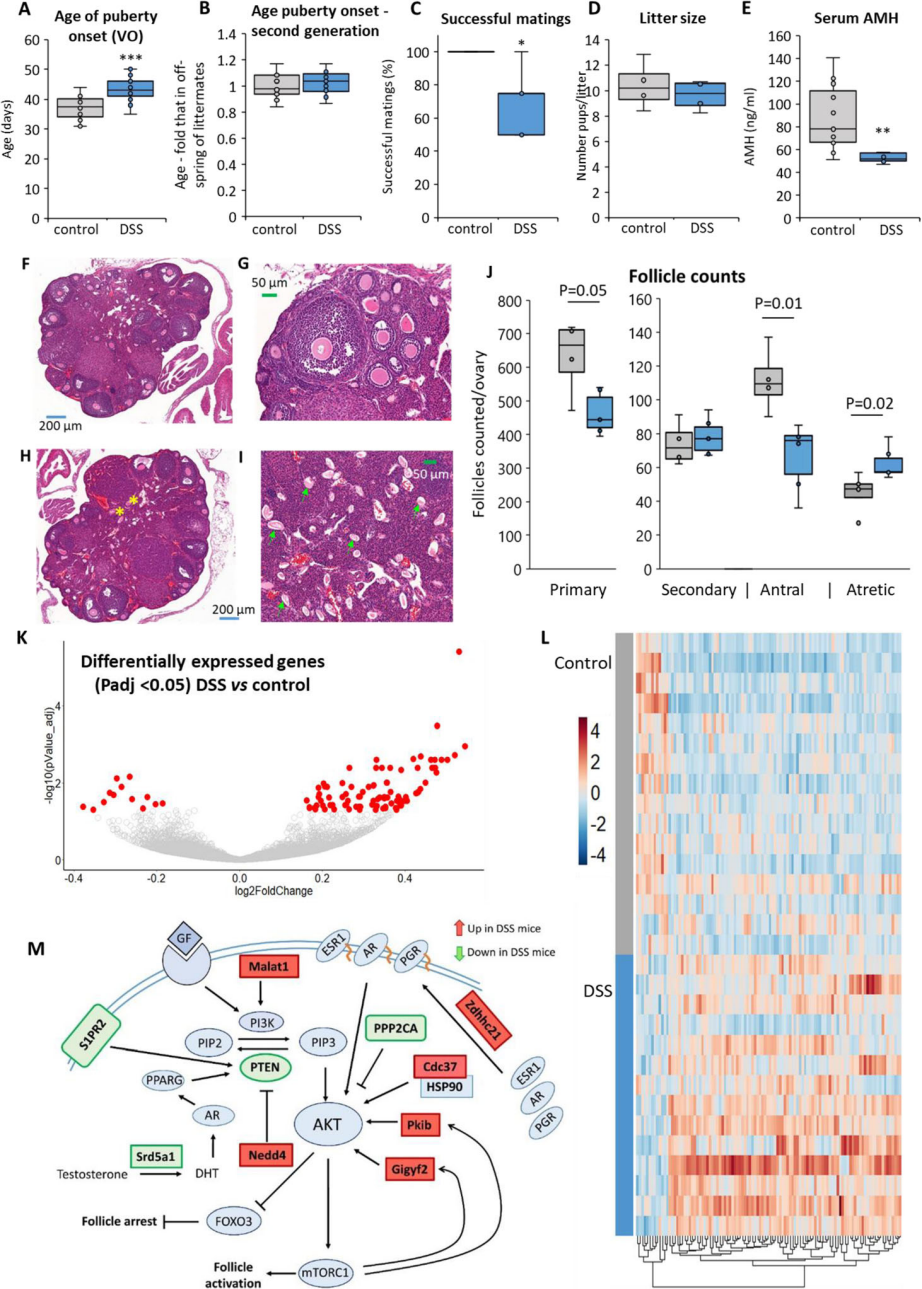
Pre-pubertal colitis in mice affects pubertal timing and ovarian function. **A** Age of vaginal opening (VO), indicating onset of puberty. ***P < 0.001; *n* = 47, 59. **B** Age of VO in second generation, relative to that in off-spring of parent littermate controls. *P* > 0.05; *n* = 20, 30. **C** Ovulatory rates (average number of successful pregnancies for each mouse) and **D** litter sizes in DSS-treated (*n* = 14 litters) and littermate control mice (*n* = 20 litters) from 5 mice in each treatment group after four encounters with the same male; *P = 0.024 (Mann-Whitney *t*-test). **E** Circulating AMH (ng/ml) in control [*n* = 11] and DSS-treated [*n* = 14] mice, **P = 0.008. **F**–**I** H&E stained ovarian histological sections from **F**, **G** control and **H**, **I** DSS-treated groups. Some atretic follicles (yellow asterisks) and *zona pellucida* remnants (green arrows) are marked. **J** Follicle counts from sections of mice ovaries (*n* = 4, *n* = 6), compared by *t*-test. **K** Volcano plot and **L** heat map of differentially expressed ovarian genes at P*adj* < 0.05 from RNA-seq analysis. **M** Signaling pathway to follicle activation, showing some of the differentially expressed genes (DEGs; *P* < 0.05). Red boxes signify upregulated genes; green boxes signify downregulated genes

In order to examine the mechanisms through which early-life inflammation-associated energetic demands affect reproductive function, we adopted a mouse model of temporary colitis in newly-weaned female mice by administration of dextran sulfate sodium (DSS). The DSS-treated mice stopped gaining weight during the latter part of the 7-day treatment and blood was evident in the feces, but they quickly recovered (Fig S1A). The treated mice had a delayed onset of puberty (as indicated by vaginal opening) by an average of 6.5 days (*P* = 1.93E−14; Fig. 1A), corresponding to ~ 1.8 y in human lifespan [34]. This delay was not inherited transgenerationally, and puberty in the female mice offspring occurred at a similar age to that in offspring of littermate controls (Fig. 1B). Notably, the treated mice had fewer successful matings than their littermate controls, although litter sizes were similar (Fig. 1C, D).

Levels of circulating anti-Müllerian hormone (AMH) were significantly lower in the DSS-treated mice (at 55 days old) than in the controls (Fig. 1E). Murine AMH levels vary along the lifespan as in humans, decreasing in adult mice as they age, correlating with numbers of growing ovarian follicles and reflecting the size of the ovarian reserve [35]. This drop in AMH levels therefore indicates a smaller ovarian reserve in the DSS-treated mice. Ovarian histological sections from the DSS-treated mice showed that they had fewer primary and antral follicles and more atretic follicles than their littermate controls (Fig. 1F–J). This is unlikely to be a direct effect of DSS on the ovaries because DSS effects are reported to be highly localized to the intestine [36], suggesting that the physiological response is probably due to the inflammation or related stress-activated pathways.

To determine the pathways responsible for the altered ovarian activity, we carried out RNA-seq transcriptome analysis. Coding and non-coding RNAs were differentially expressed: 92 were upregulated and 13 downregulated (P*adj* < 0.05; Fig. 1 K, L). Pathway analysis (Fig S2) of the differentially expressed genes (*P* < 0.05) revealed enrichment specifically for oocyte-meiosis (false discovery rate [FDR] 1.9−E02). Genes in the Hippo (FDR 4.3-E01) and PI3K-Akt signaling pathways that stimulate the recruitment of ovarian follicles [37, 38] were also enriched (Fig S2), and the expression of PTEN which represses this pathway was reduced (Fig. 1M).

### Integrating ovarian differential gene expression in the mouse model with differential methylation signatures in Bangladeshi women who grew up in Bangladesh or in the UK

With the aim of identifying genes associated with differential methylation patterns in women who had faced similar childhood immune challenges, we carried out methylation analysis of buccal DNA collected from Bangladeshi women living in London who had grown up either in Bangladesh or the UK. We reasoned that any such genes that were also differentially expressed in the mouse ovaries might reflect environmentally responsive, epigenetically regulated genes that might be involved in ovarian function. We found distinct methylation signatures in these two groups of Bangladeshi women: 17,004 CpG sites had a mean methylation difference > 20%, most of which (14,509) mapped to “open sea” regions of the genome; a smaller number (2423) were associated with “shores” and “shelves” and 72 mapped within CpG islands. Pathway analysis revealed, as in the mouse RNA-seq study, that genes associated with differentially methylated CpGs were enriched in the Hippo (FDR 6.99E−12) and PI3K-Akt (FDR 2.83E−07) signaling pathways (Fig S3A-C). We confirmed by targeted bisulfite sequencing the differential methylation associated with some of these genes, focusing on those with known functions in fertility. These included elevated methylation levels in CpG islands associated with *FZD1* and *RUNX3*, both of which encode proteins that regulate ovarian folliculogenesis [39, 40], and also *RASAL3*, which controls a magnitude of inflammatory responses [41] and has been linked specifically to inflammatory bowel disease [42] (Fig S3D).

**Fig. 2:**
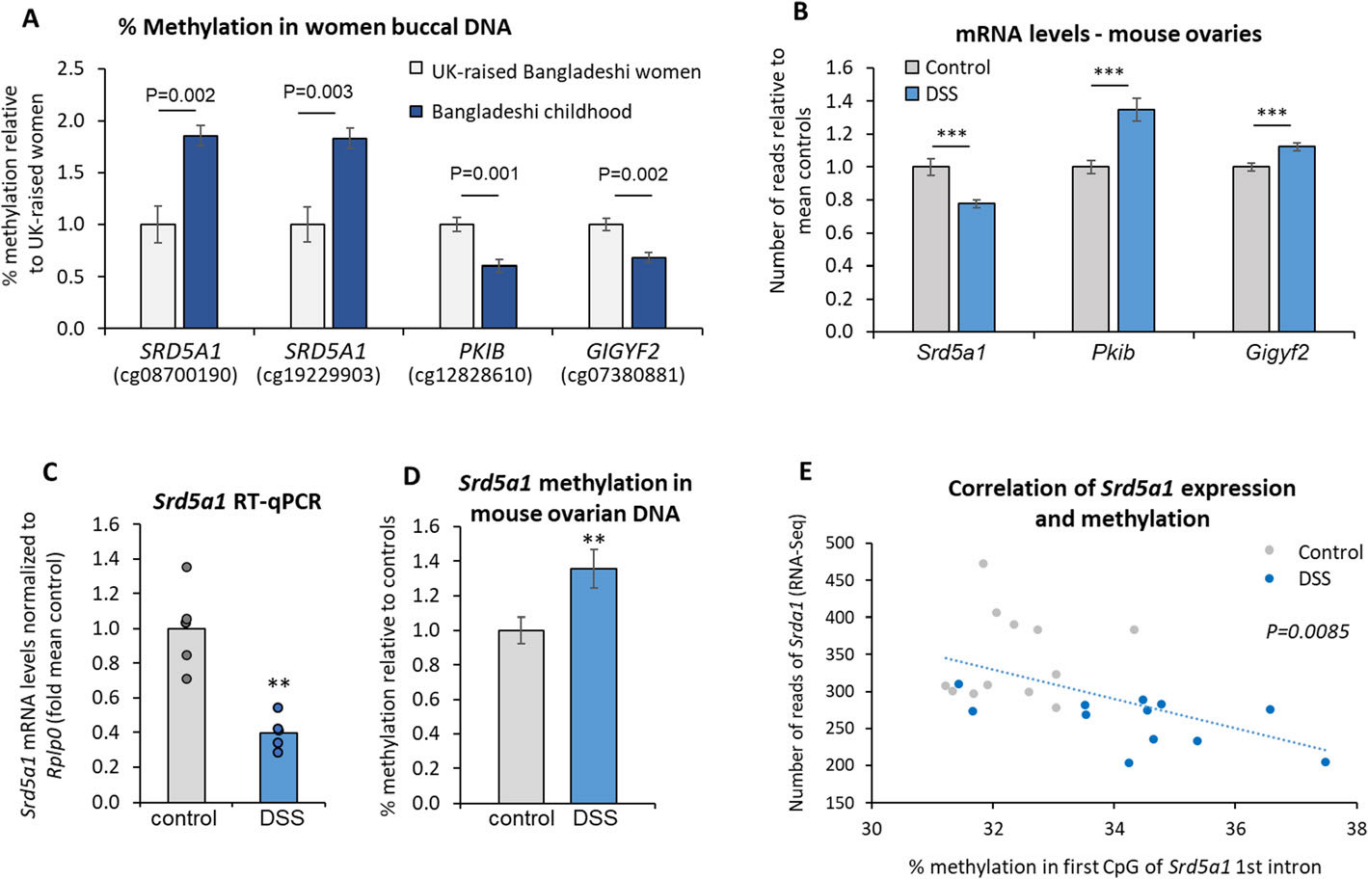
Srd5a1 is downregulated and hypermethylated in mice and women following early-life challenge. **A** Differentially methylated regions associated with three genes (Illumina EpicMethylation sites) in buccal DNA of Bangladeshi women who grew up in Bangladesh (*n* = 16) or UK (*n* = 13); mean ± SEM. **B** The mRNA levels of the same three genes in control (*n* = 16) and DSS-treated (*n* = 14) mice ovaries from the RNA-seq analysis; ***P*adj* < 0.001; mean ± SEM. **C** Confirmatory qPCR analysis of the *Srd5a1* mRNA levels (*n* = 5), **P = 0.007; showing means with individual data points. **D** Levels of CpG methylation in the 5’region of the *Srd5a1* first intron (corresponds with first site in Fig. 2A: see Fig S4), in control (*n* = 24) and DSS-treated (*n* = 29) mice ovaries, mean ± SEM shown relative to controls; **P = 0.015 (Mann-Whitney *t*-test). **E** Correlation between the levels of *Srd5a1* mRNA (from RNA-seq analysis) and methylation measured in the same samples; *P* = 0.0085

Of the genes associated with differentially methylated regions in the women’s DNA, 13 had altered methylation at one or more CpGs that correlated with the differential expression of the orthologous gene in the mouse ovaries (Table S1). These genes included *PKIB* and *GIGYF*, which were associated with less methylation in the women who had experienced childhood in Bangladesh (Fig. 2A), while their expression was upregulated in the ovaries of the DSS-treated mice (Fig. 2B). Both genes encode proteins that activate AKT (Fig. 1M). In contrast, *SRD5A1,* whose expression was reduced in the mouse ovaries, was more methylated in the DNA of these women (Fig. 2A–C).

### SRD5A1 is hypermethylated at a corresponding location in the first intron of mice and women following the early-life challenges

In contrast to the other genes in this overlapping dataset, whose relevance to function of the reproductive axis was less clear, the increased methylation (in women) and reduced expression (in mice) of *SRD5A1* was striking, given that this gene encodes the steroidogenic enzyme, 5α reductase-1. This enzyme catalyzes conversion of testosterone to the more potent androgen, dihydrotestosterone (DHT), and also of progesterone to dihydroprogesterone, and deoxycorticosterone to dihydrodeoxycorticosterone. Reduced expression of 5α reductase-1 or its steroid products has been observed in various studies on adult rodents and humans after early-life stress [33, 43–46], and in the ovary, DHT is reported to repress follicle recruitment [31, 32]. We thus hypothesized that the increased methylation of *SRD5A1* might lead to reduced activity of 5α reductase-1 and that this is responsible for some of the altered reproductive characteristics.

To test this hypothesis, we first examined the *Srd5a1* promoter in the mouse model and found it completely unmethylated in the ovaries of both DSS-treated and control mice (Fig S4A). We therefore examined DNA methylation at the region corresponding to that differentially methylated in the women’s DNA, located in the gene’s first intron (Fig S4B), and found it to be significantly more methylated in the ovaries of the DSS-treated group than in the controls (Fig. 2B). First introns commonly contain enhancer elements [47], and the start of the *Srd5a1* first intron comprises a conserved region that carries marks of a transcriptional enhancer (Fig S4B) [48]. A likely regulatory function for this *locus* is further supported by the presence of a single nucleotide polymorphisms (SNP) which is associated with age at natural menopause, and two other significant SNPs in high linkage disequilibrium with this trait [49]. Accordingly, there was strong correlation between levels of *Srd5a1* mRNA expression and CpG methylation at this site (Fig. 2E).

### Upregulation of Srd5a1 by estradiol is blunted by anti-inflammatory cytokines

**Fig. 3:**
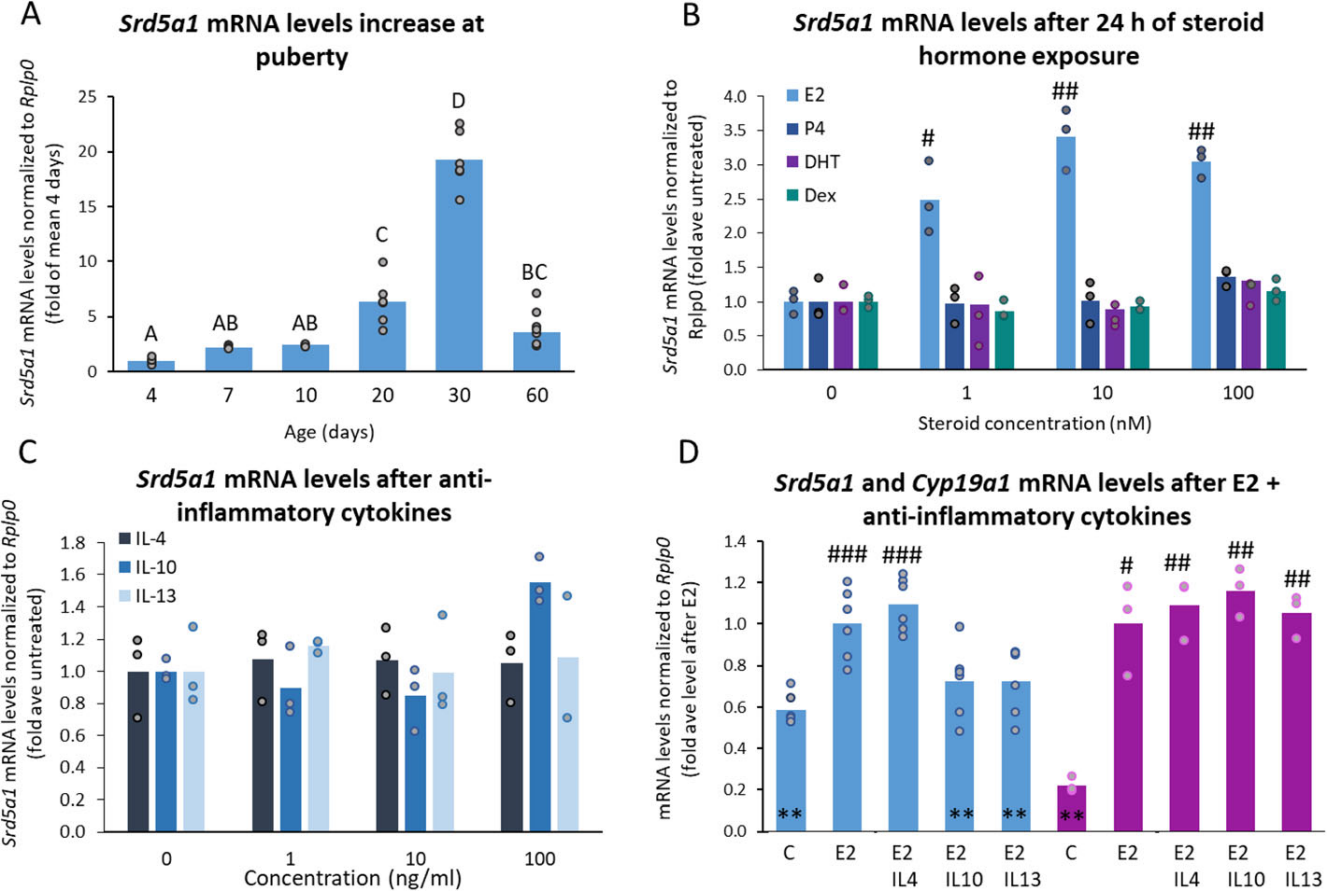
The upregulation of Srd5a1 by estradiol is blunted by anti-inflammatory cytokines. **A** *Srd5a1* mRNA levels in ovaries of mice of various ages (*n* = 3–7). Groups sharing same letter: *P* > 0.05 (ANOVA, Tukey-Kramer *t*-test). **B**–**D** In ovarian KK-1 cells: **B** *Srd5a1* mRNA levels (*n* = 3) after exposure to estradiol (E2), progesterone (P4), dihydrotestosterone (DHT), or dexamethasone (Dex). For E2-treatments, #*P* < 0.05; ##*P* < 0.01 compared to controls, otherwise *P* > 0.05. **C** *Srd5a1* mRNA levels after cytokine exposure in KK-1 cells (*n* = 3). **D** Also in KK-1 cells, *Srd5a1* (*n* = 6) and *Cyp19a* (*n* = 3) mRNA levels after E2 alone (10 nM) or with cytokine (100 ng/ml); #*P* < 0.025, ##*P* < 0.01, ### *P* < 0.001 vs control; **P < 0.02 vs E2; where not marked *P* > 0.05. All graphs show mean with individual data points

To determine how early-life events might affect *SRD5A1* expression, we first examined its levels during normal development. Comparison of *Srd5a1* mRNA levels in ovaries from untreated mice of different ages revealed that they increase dramatically over the course of sexual maturation (Fig. 3A), indicating a possible role for hormones activated at puberty in regulating *Srd5a1* expression. We therefore exposed ovarian KK-1 mouse granulosa cells to various gonadal steroids, which revealed a stimulatory effect of estradiol (E2) on *Srd5a1* mRNA levels, while neither DHT, progesterone nor dexamethasone had any notable effects (Fig. 3B, Fig S5). This suggests that the rise in *Srd5a1* expression at the time of puberty is likely due to the increase in E2 levels and that the early life adversity either functions independently to repress *Srd5a1* or might interfere with this activation.

DSS-treatment is expected to induce a general stress response, elevating levels of corticosterone (the main corticosteroid in mice) and anti-inflammatory cytokines [50]. However, corticosterone did not seem to be responsible for the drop in *Srd5a1* expression in the DSS-treated mice, given that dexamethasone (synthetic corticosteroid) did not affect *Srd5a1* mRNA levels in the KK-1 cells (Fig. 3B), though a control gene, *Rasd1*, did respond (Fig S5). We therefore tested, also in KK-1 cells, whether the anti-inflammatory cytokines, IL-4, IL-10, or IL-13 exerted any effect on *Srd5a1* expression. These cytokines alone did not reduce basal *Srd5a1* levels (Fig. 3C), but when given together with E2, both IL-10 and IL-13 blocked the E2-stimulatory effect on this gene without affecting the E2 upregulation of the *Cyp19a1* control gene (Fig. 3D). Thus, early life events involving an increase in IL-10 and/or IL-13 at the time that E2 levels normally increase might dampen the stimulatory effect of E2 on *Srd5a1* expression. This could explain the significance of exposure to such stressors at this developmental stage.

### 5α reductase-1 regulates the central control of reproduction and pubertal onset

**Fig. 4:**
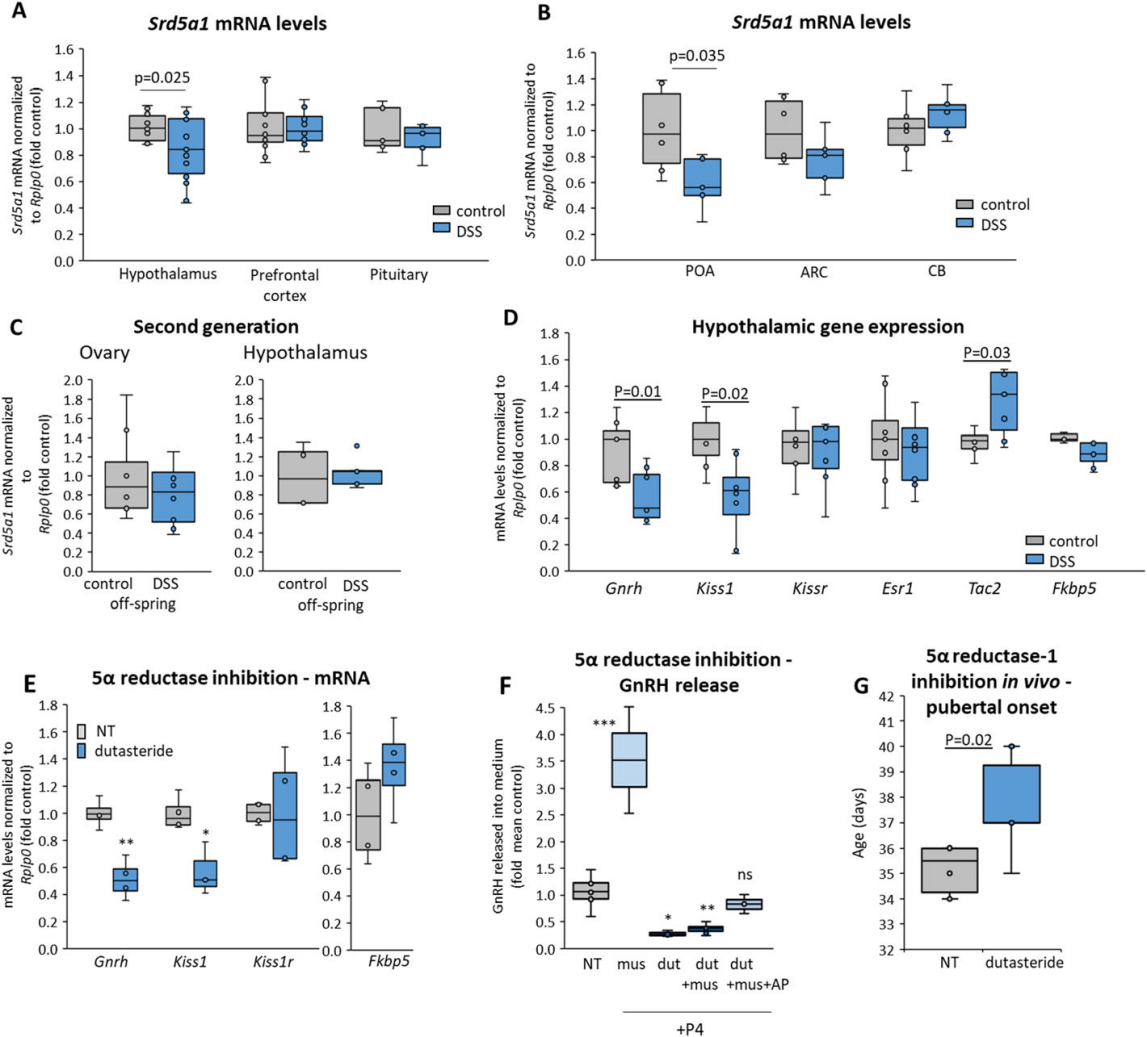
5α reductase-1 regulates the central control of reproduction and pubertal timing. *Srd5a1* mRNA levels were measured in **A** whole hypothalamus (*n* = 14), prefrontal cortex (*n* = 15, 14) and pituitary (*n* = 7) of control and DSS-treated mice or **B** preoptic area (POA: *n* = 6, 5) and arcuate nucleus (ARC: *n* = 6, 5) of the hypothalamus, and the cerebellum (CB: *n* = 6). **C** *Srd5a1* mRNA levels in the ovary (*n* = 8) and whole hypothalamus (*n* = 4, 5) of female off-spring of DSS-treated mice and their littermate controls. **D** The mRNA levels of genes encoding reproductive regulatory factors were measured in the whole hypothalamus of the DSS-treated and control mice, with *Fkbp5* as an indicator for stress (*n* = 7 or 8). **E**, **F** The effect of 5α reductase inhibitor, dutasteride in GT1-7 GnRH neuronal cells on **E** gene expression (*n* = 4 or 3) and **F** GnRH release, in which some cells were also exposed to the GABA_A_R agonist, muscimol, alone (*n* = 2), with dutasteride (*n* = 7) or together with allopregnanolone (AP) (*n* = 3), all in the presence of AP precursor, P4 (for NT *n* = 7, for dutasteride alone *n* = 3). **G** Age of VO following dutasteride: female mice and their control littermates were given dutasteride (or vehicle) in the diet each day after weaning, and checked daily for VO (*n* = 6). For all, significant differences are shown for comparisons with control mice or untreated cells, otherwise *P* > 0.05

5α reductase-1 is widely expressed, including in the brain where it catalyzes synthesis of neurosteroids [33, 43, 46], and some of these activate GnRH neurons at puberty via GABA_A_ receptors [51–54]. We therefore considered that, if *SRD5A1* expression is downregulated also in the brain following early-life stress, this could play a role in delaying pubertal onset. The *Srd5a1* mRNA levels were found to be reduced in the hypothalamus of the DSS-treated mice, but not in the prefrontal cortex or the pituitary (Fig. 4A). In additional mice, separation of the hypothalamus into distinct regions revealed its reduced expression in the pre-optic area (Fig. 4B), which contains most of the neurons that control reproduction. A possible connection between delayed pubertal onset and *Srd5a1* reduction was supported by the fact that the second generation mice had neither a delay in pubertal onset (Fig. 1B) nor reduced *Srd5a1* mRNA levels in the hypothalamus and ovaries (Fig. 4C) compared to controls.

To test whether the drop in *Srd5a1* expression in the hypothalamus correlated with reduced expression of genes encoding GnRH and other key factors that regulate the reproductive axis, we measured also their expression levels. *Gnrh* mRNA levels were significantly lower in the DSS-treated mice than in their litter-mate controls, as were those of *Kiss1* (Kiss1 regulates *Gnrh* expression and release via binding its receptor on GnRH neurons: Fig S6); notably, mRNA levels of *Tac2* (the mouse ortholog of human *TAC3*) were elevated, while those of Kiss1 receptor, *Kiss1r*, and estrogen receptor, *Esr1*, were unaltered (Fig. 4D). The expression of *Fkbp5,* which is highly stimulated by corticosteroids, was not different in these mice (Fig. 4D), indicating that the mice were not suffering chronic stress, and that the genes that regulate the reproductive axis were affected specifically.

Having established the correlation between the drop in *Srd5a1*, *Gnrh*, and *Kiss1* expression in the mice whose puberty was delayed, we went on to test causality by examining the effects of 5α reductase-1 inhibition on these genes. In GT1-7 GnRH neuronal cells, exposure to dutasteride which inhibits 5α reductase activity [55, 56] led to a reduction in expression of both *Gnrh* and *Kiss1*, while *Kiss1r* and *Fkbp5* levels were unaffected (Fig. 4E) (*Kiss1* and *Kiss1r* mRNA and protein have been reported previously to be expressed in this cell line [57–60]). Thus, the actions of 5α reductase-1 appear to play a role in regulating the expression of Kiss1 and GnRH.

Some of the neurosteroids catalyzed by 5α reductase-1, including allopregnanolone, have been reported to augment GnRH release at puberty through activating the stimulatory GABA_A_ receptor [53, 54, 61]. We therefore looked to see if GnRH release was also affected by dutasteride-mediated 5α reductase-1 inhibition. We found that the stimulation of GnRH release by a GABA_A_ agonist, muscimol, was completely blocked by the dutasteride; however, levels were restored to those of controls by the addition of allopregnanolone (Fig. 4F). These experiments indicate that 5α reductase-1 activity is required for GnRH release and that at least part of this effect might be mediated via allopregnanolone.

After seeing that the DSS treatment reduces hypothalamic expression of *Srd5a1*, and that 5α reductase-1 is required for GnRH synthesis and secretion, we went on to examine the impact of a reduction in this enzyme’s activity on the timing of pubertal onset in vivo*.* Daily administration of dutasteride to young female mice, starting soon after weaning, delayed vaginal opening by an average of 3–4 days compared to sham-treated litter-mate controls (Fig. 4G); this corresponds to an estimated ~ 1 year in human lifetime [34]. Thus, we have established that a drop in 5α reductase-1 expression can drive a delay in pubertal onset.

## Discussion

Numerous studies have illustrated variation in human reproductive phenotypes from populations inhabiting different environments [11, 12, 62–67], and these have often been interpreted as adaptive responses aiming to optimize reproductive success [6, 7, 68–70]. These studies have provided fascinating insights into the connection between the early-life environment, pubertal timing, and adult reproductive function and demonstrate plasticity particularly in response to early-life nutrition [4, 70–73], social stress, and other forms of physical and psychological adversity [63, 74–80]. However, the inaccessibility of hypothalamic-pituitary-gonadal (HPG) tissues in healthy human subjects, and reliance on proxy tissues for epigenome-wide association (EWAS) studies, does not allow this type of study to establish causality or necessarily elucidate the mechanisms responsible for the change in phenotype [5]. DNA methylation, which is often seen as a marker of plasticity, can be the mechanism responding to and driving altered phenotypes, but confirmation of this requires study of the functional tissues.

Here, by consolidating data on differential methylation in the Bangladeshi women who had grown in Bangladesh (more pathogenic environment) or the UK, together with differences in ovarian gene expression in a mouse model of prepubertal immune challenge, we identified the downregulation in *SRD5A1*. Not only was this drop in *Srd5a1* expression correlated with elevated methylation at a transcriptional enhancer in the mice, but the same genomic region had higher methylation levels in buccal DNA of Bangladeshi women who had grown up in Bangladesh as opposed to the UK. The fact that, like these women [10, 13, 81], the mouse model had smaller ovarian reserves, later pubertal onset and possibly lower ovulatory rates, and that this gene encodes a widely-expressed steroidogenic enzyme with key roles in the HPG axis, led us to hypothesize that the *SRD5A1* downregulation might mediate the effect of the early-life events on these characteristics, and the mouse model enabled us to examine its role in the central control of reproduction in vitro and in vivo.

In the ovary, 5α reductase-1 catalyzes the conversion of testosterone to DHT which inhibits follicle activation through decreasing cyclin D2 expression and inducing cell cycle arrest [31], as well as via activation of PTEN which represses PI3/AKT signaling [32]. Thus, the drop in 5α reductase-1 expression observed in the mouse model would be expected to facilitate oocyte exit from the follicle pool, in accordance with the ovarian histology and lower AMH levels in the mice, and the lower AMH levels and earlier menopause in the Bangladeshi women who had grown up in Bangladesh [12]. Notably, DHT also activates progesterone production [82], so the drop in 5α reductase-1 expression would also explain the lower progesterone levels noted in these women [10]. Our study thus provides an underlying mechanism for this plasticity in ovarian function, involving the epigenetically mediated downregulation of *SRD5A1.*

A previous study found that delayed puberty following DSS treatment in mice was associated explicitly with inflammation and increased cytokine levels, and was not due merely to weight loss, and that it occurred irrespective of serum leptin and corticosterone levels [30]. Studies in human populations have also reported a correlation between inflammatory markers, progesterone levels and ovarian suppression [63], while maternal inflammation and ratios between specific cytokines was found to be associated with fetal growth rates [83]. The energetic costs of immune challenges arising from childhood in Bangladesh or prepubertal colitis in mice likely force trade-offs with other physiological systems [84, 85]. This might reflect an adaptive strategy to optimize reproductive success over the life-course and could explain the women’s shorter reproductive lifespans [5–7].

The epigenome is highly responsive to various metabolic, cellular, and physiological signals [25, 26], and the immediate pre-pubertal period is clearly a particularly sensitive period for moderating *SRD5A1* expression given that it is normally upregulated at this time, as seen also in other developmental genome-wide screens [86]. We found that anti-inflammatory cytokines, activated by the stress response, block the stimulatory effects of E2 on *Srd5a1* mRNA levels in ovarian cells, while other studies have reported decreased expression of 5α reductase-1 and/or its metabolites long after experiencing various physiological and psychological stressors, with the effect being most pronounced when the stress was experienced at a young age [33, 43–46]. These findings support a role for the epigenetic modification of *SRD5A1* during childhood in response to a demanding or stressful environment, leading to the re-programming of reproductive function and perhaps also of other major endocrine axes. Our results are also in tune with other studies implicating androgens as playing a crucial role in developmental plasticity and male reproductive strategies [87, 88].

In the hypothalamus, the role of 5α reductase-1 in activating the reproductive axis and pubertal onset involves the synthesis of neurosteroids, including allopregnanolone which regulates GnRH levels [51, 61]. We found that 5α reductase-1 inhibition represses upstream regulators of the reproductive axis and delays pubertal onset, providing an additional mechanism by which epigenetic regulation of *SRD5A1* would alter the reproductive phenotype. However, 5α reductase-1 also catalyzes the production of additional neurosteroids, some of which regulate the growth and stress response axes [33, 51, 89], and are associated with insulin resistance, metabolic disease, polycystic ovarian syndrome, and inflammation [90]. In the face of early-life adversity, altered epigenetic regulation of *SRD5A1* in the hypothalamic control center could therefore perhaps affect any of the major endocrine axes in response to changing environmental conditions. The resulting changes in 5α reductase activity levels might mediate the ability to allocate resources differentially to growth, reproduction, and homeostasis as described in life-history theory [91–93], as well as the trade-offs between early life development and the risks for metabolic disorders later in the life-course that have been reported [7].

Mid-childhood has received less attention as a time of epigenetic programming than the gestational or immediate post-natal developmental periods. However, considerable changes in methylation occur at this time [94, 95] which have been associated particularly with changes in the epigenetic state of genes relevant to cell growth and the immune system [96]. Moreover, this is a time of dramatically changing steroid levels and neuronal development which would be expected to be sensitive and responsive to various external signals [69, 97]. In fact, this period in development has been suggested to comprise a “switch point” in the development of life history strategies [9] in which long-term trajectories might be established, including for resource utilization in accordance with conditions of the early life environment [5]. Our findings support the notion that this pre-pubertal stage is indeed a window of sensitivity for determining some of these strategic trajectories relating to adult reproductive function.

## Conclusions

The methylation-mediated regulation of *SRD5A1*, which encodes a factor that moderates reproductive function, and the down-stream response to its lower levels that we have described, appear to play a key role in mediating plasticity of reproductive function. It could be speculated that the altered methylation status of this gene, as opposed to *Gnrh* or *Kiss1*, might be subtle enough for the reproductive system both to sense and tolerate without implementing compensatory mechanisms to overcome it, allowing *SRD5A1* to function in this context. Such epigenetically driven reprogramming could explain some of the diversity in reproductive characteristics and how they are shaped by the early-life environment. Although such adaptations might be beneficial for an individual’s fertility, the altered reproductive phenotype presents health consequences, as timing and duration of reproductive events influence risks for steroid- and age-related disease [7, 98]. Understanding these epigenetic processes reveals new biomarkers and pathways that might be manipulated to mitigate health issues caused by these adaptive strategies, while also shedding light on disorders such as polycystic ovarian syndrome for which an epigenetic basis has already been implicated [99–101]. At present, clinicians follow normative models of reproductive function such that phenotypes outside these ranges (primarily derived from healthy, well-nourished Western populations) are usually defined as pathological rather than reflecting normal variation [102]. Perspectives derived from evolutionary biology and reproductive ecology could expand viewpoints among health professionals and lead to greater appreciation of reproductive variability, as well as the sources of that variability.

## Methods

### Mice

All mice (inbred transgenic C57BL/6) were held and handled humanely, after protocol approval, and in accordance with Institutional Animal Care and Use Committee (IACUC) guidelines. For DSS-treatments, upon weaning (22–23 days old: approximately equivalent to 6–6.5 years in human age [34]), female mice from each litter were divided randomly into two groups to provide littermate controls for all experiments. After ~ 2 days of recovery, the mice were ear-marked and weighed, and one group received 3% dextran sulfate sodium (DSS: 35–50 kDa, MP Biochemicals) in the drinking water for 7 days. The DSS water was changed every 2–3 days, and the mice were weighed each day for at least 16 days. All mice were observed daily for signs of vaginal opening (VO) which reflects sexual maturation due to increased ovarian activity and estradiol levels and is commonly used as a proxy for pubertal onset [103]. In order to assess the impact of this treatment on ovulatory rates and on the second generation, a single male was housed with the DSS-treated and littermate control female mice (aged 2.5–7 months). For harvest of brain tissue, the brains were removed and whole hypothalamus or prefrontal cortex isolated into 1 ml TRIzol for RNA extraction. For isolation of specific regions (preoptic area, arcuate nucleus or cerebellum), brains were transferred into a brain matrix (RWD-800-00149-00) for coronal sectioning following isolation of each region from the relevant section, as determined using Allen Brain Atlas. All tissues were collected from females (aged 50-100 days old) in estrous, verified by cytological smears, to ensure that any differences in gene expression were not due to the cyclical changes in hormone levels. Blood was collected in 55-day-old mice by cardiac puncture at the time of sacrifice, and circulating AMH levels were measured by enzyme-linked immunosorbent assay (ELISA: Ansh labs, Webster, Texas) according to the manufacturer’s protocol, after dilution of all samples × 20.

For the administration in vivo of dutasteride (a drug approved by the Food and Drug Administration to reduce androgen effects by inhibiting 5α reductase activity), female mice from each litter were marked, weighed and divided into two groups upon weaning. A transparent plastic separator (kindly given by Madaf Plazit Packaging) was inserted into each cage, with one mouse in each half. Dutasteride (SML1221, Sigma) was dissolved in oleic acid (O1383, Sigma) at 15 mg/ml, and added to a ~ 60 mg piece of enriched diet pellet (D12451i, Research Diets). Immediately after separation, each mouse received the dutasteride-treated diet (~ 13 μg/g BW, a dose shown previously to affect 5α reductase activity in the brain [104]) or a similar amount of vehicle-treated diet for controls. The pellet was consumed fully within a few minutes, and the separator was then removed. The treatment was repeated daily, and mice were weighed and observed daily for signs of VO.

### Histology and follicle counts

Ovaries were harvested from the mice at ~ 60 days and were fixed with 4% paraformaldehyde for 4 h before transferral to 70% ethanol. Paraffin embedding, sectioning (4 μm), and hematoxylin and eosin (H&E) staining were carried out at the Biomedical Core Facilities at the Rappaport Faculty of Medicine, Technion-Israel Institute of Technology. Identification of follicle stage (using CaseViewer software) and counting were performed (as in [105, 106]), while blind to the treatment group. In short, every fifth section per ovary was analyzed, and the follicular stage was determined by size and morphological characteristics: primary follicles containing a single layer of cuboidal granulosa cells; secondary follicles showing more than one layer of granulosa cells but no antrum, and antral follicles containing an antral space. Atretic follicles were identified based on the presence of *zona pellucida* remnants, stained bright pink. Secondary and antral follicles were counted only if a nucleus was present, and the atretic follicles, which vary considerably in size, were counted every 8th stained section, to avoid counting the same follicle twice. The “follicle counts” presented comprise the number of follicles at each of these stages counted in each ovary using this approach.

### Quantitative PCR, transcriptome, and methylation analysis

RNA was isolated using TRIzol, DNase I-digested and cleaned using R1014 RNA Clean & Concentrator-5 kit (Zymo Research), cDNA synthesized using the qScript Flex cDNA kit (95049 Quanta) using oligo dT, and real-time quantitative PCR (qPCR) was carried out using the PerfeCTa SYBR Green FastMix (Quanta), both as previously reported [107], using primers listed in Table S2. Amplicon levels were quantified using standard curves and normalized to levels of *Rplp0.*

For transcriptome analysis, RNA was extracted from the mice ovaries (mice were 67 ± 3.7 or 72 ± 4.5 days old respectively at sacrifice) and purified as above, and sequenced by CEL-seq, using Illumina HiSeq 2500 at the Technion Genome Center (as in [24]). FastQC was used for quality control and the reads were mapped by TopHat algorithm to mm10 genome assembly. HTSeq-count was used to count the reads, and the normalization of raw counts and differential expression were calculated using DESeq2 in R platform, with P*adj* using Benjamini and Hochberg correction for false discovery. Pathway analysis was performed using the Database for Annotation, Visualization and Integrated Discovery (DAVID).

DNA was extracted from the mouse tissues using TRIzol (after upper phase was taken for RNA extractions), and the genomic DNA cleaned with the Quick-DNA Miniprep Plus Kit (D4068; Zymo), before bisulfite conversion using the EZ-DNA Methylation-Gold Kit (D5005 Zymo), and two rounds of PCR-amplification (nested, with outer and inner primers: Table S2) using Red Load Taq Master (Larova). After purification of the amplicons (PCR purification kit; Qiagen) and cloning into pGEM-T-easy, inserts from 7 to 8 randomly selected clones were sequenced and analyzed as previously [108]. Subsequently, the region in the first intron homologous to that differentially methylated in the human samples was amplified and cleaned as above. Additional rounds of PCR were then performed using KAPA HiFi HotStart Ready mix (Roche), initially with primers containing the adaptors (Universal adaptors; Illumina) and subsequently another 8–12 PCR cycles with the specific primers (Illumina Nextera XT index kit); samples were cleaned with PCR purification kit (Qiagen) between each stage. These libraries, after addition of 50% Phi-X, were then deep-sequenced by 150 bp paired-end sequencing on Mi-seq (Illumina), at the Technion Genome Center.

### Human methylation analysis

We sampled first- and second-generation British-Bangladeshi women representing participants in our numerous previous studies from which we have reported rich quantitative and qualitative data on diet, lifestyles, migration, and demographic histories [10, 12, 13, 28, 29, 109]. The samples analyzed here were from women aged 21–35 years, recruited in London through community contacts using snowballing techniques. The first group comprised women who were born in Bangladesh and moved to the UK when aged over 16 years. The second group comprised women who were second-generation British-Bangladeshis, born in the UK to Bangladeshi migrant parents. Protocols for human data collection were approved by the Ethical Committee of the Department of Anthropology, Durham University. Women gave informed consent to participate in the study, and the data were anonymized at source.

Buccal swabs were collected with iSWAB (Mawi DNA Technologies) and genomic DNA isolated using the DNeasy Blood & Tissue Kit (Qiagen). Genome-wide DNA methylation data acquisition was carried out on the Infinium MethylationEPIC BeadChip platform (Illumina) and performed by Tepnel Pharma Services, UK using “Bangladeshi childhood” (*n* = 16) and “UK childhood” (*n* = 13) DNA samples, which passed the quality control checks. Multidimensional scaling (MDS) plots indicated that no significant batch effects were skewing our MethylationEPIC BeadChip data sets. The data were processed with the Bioconductor/minfi package. CpG probes associated with known SNPs were removed, as were those with a detection probability of < 0.01. Probes on both X and Y chromosomes were retained. Methylation beta values (0–1) were normalized by SWAN. Dmpfinder/minfi was applied to determine probes with significantly differentially methylation levels between the “Bangladeshi-childhood” and the “UK-childhood” groups. FDR was set at < 0.05. The pathway analysis utilized NIPA, a tool that performs enrichment tests by hypergeometric statistics (https://github.com/ADAC-UoN/NIPA/).

For validation of the array data, targeted bisulfite sequencing was conducted on a subset of the samples used to generate the MethylationEPIC BeadChip data (chosen on the basis of their DNA quality and concentration) via amplicon sequencing of 10 CpG sites using the MiSeq system (Barts and the London School of Medicine and Dentistry, GenomeCentre). The interrogated CpGs were cg25470148 (chr1:25257931) lower-strand, *RUNX3*; cg16696646 (chr17:19861616), lower-strand *AKAP10*; cg26916966 (chr17:40274524), upper-strand, *KAT2A*; cg07357279 (chr17:43318735), upper-strand, *FMNL1*; cg01062942 (chr19:15568935), upper-strand, *RASAL3*; cg08470875 (chr2:26401718), upper-strand, *FAM59B*; cg08700190 (chr5:6636046), upper-strand, *SRD5A1*; cg08198075 (chr6:123033536), upper-strand, *PKIB*; cg12914114 (chr6:170687002), lower-strand, *FAM120B*; cg01480180 (chr7:90896329), lower-strand, *FZD1*. For each interrogated genomic region, > 100 sequencing reads were obtained. Primers are given in Table S3.

### Cell culture

The murine KK-1 granulosa cell line (a gift from Ilpo Huhtaniemi, Imperial College, UK) was cultured as reported [110], maintained at 37 °C with 5% CO_2_ at 30–80% confluency, passaging 2–3 times a week. The media was changed to the same media but with charcoal-stripped fetal bovine serum (FBS), 24 h before and during the treatments with either 1–100 nM of the steroids (Sigma) for 24 h, or anti-inflammatory cytokines: IL-4, IL-10, or IL-13 (1-100 ng/ml for 24 h), alone or before addition of E2. Alternatively, the GT1-7 mouse hypothalamic GnRH neuronal cell line was cultured with high glucose Dulbecco's Modified Eagle Medium containing 10% FBS, 1% penicillin-streptomycin, sodium pyruvate, and sodium bicarbonate, maintained at 37 °C with 5% CO_2_ at 50–90% confluency, passaging 1–2 times a week. Cells are tested regularly for mycoplasma and identity authenticated through hormone responsiveness. For mRNA measurements, the GT1-7 cells were cultured in charcoal-stripped FBS medium for 24 h before some were exposed to dutasteride (5 μM) for 24 h. Cells were then harvested for RNA extraction and qPCR analysis as before.

For analysis of GnRH release, cells (in 6-well plates) were washed twice in medium without FBS, incubated in the same medium, and some exposed to dutasteride (10 μM). After 30 min, progesterone (P4; 2 μM) was added for 5 h, and then muscimol (100 μM) or/and AP were added for 1 h. The medium was collected into 1.5 ml tubes, centrifuged for 2 min at 3000*g* and kept at − 80 °C for measurement of GnRH in the supernatant by ELISA (Phoenix Pharmaceuticals, catalog # EK-040-02 CE), according to the manufacturer’s protocol.

### Statistical analysis

All data are from multiple biological repeats (*n*-value) which were assayed individually. Results are shown as box plots (whiskers show minimum and maximum values, boxes represent 25–75% data ranges, horizontal lines within boxes indicate the median values); for groups of *n* < 6, outliers are shown so all points are visible. Alternatively, data are shown as individual values or as mean ± SEM. Statistical analysis for parametric data was using a Student’s *t*-test (two-tailed), and differences considered significant at *p* ≤ 0.05, or alternatively one-way analysis of variance (ANOVA), followed by the Tukey-Kramer or Bonferroni *t*-test for multiple comparisons. Methylation analysis (% methylation) and mating success (% successful pregnancies) utilized Mann-Whitney non-parametric t-test.

## Supplementary Information

**Additional File 1: Tables S1-S3. Figures S1-S6**. **Table S1.** Genes associated with women’s methylation which correlated with mice expression. **Table S2.** Primers list (mice). Table S3. Primers list (human). **Fig. S1.** Weight change in DSS treated and control mice. **Fig. S2.** Pathway analysis of differentially expressed genes in mice ovaries. **Fig. S3.** Human Methylation analysis. **Fig. S4.** Differential SRD5A1 methylation. **Fig. S5.** Positive controls for steroid treatments in KK-1 cells. **Fig. S6.** Schematic illustration of the core biological pathways.

## Data Availability

Data are available at NCBI's Gene Expression Omnibus (GEO), GSE133355 (human DNA methylation data) [111], and GSE133633 (mouse RNA-seq data) [112].

## Abbreviations

DEG: Differentially expressed gene
DHT: Dihydrotestosterone
DSS: Dextran sulfate sodium
E2: Estradiol
ELISA: Enzyme-linked immunosorbent assay
FBS: Fetal bovine serum
FDR: False discovery rate
GnRH: Gonadotropin-releasing hormone
IACUC: Institutional Animal Care and Use Committee
P4: Progesterone
qPCR: Quantitative polymerase chain reaction
SNP: Single nucleotide polymorphisms
VO: Vaginal opening
