# Supplementary material for: Epigenetic regulation of 5α reductase-1 underlies adaptive plasticity of reproductive function and pubertal timing
Source: BMC Biol. 2022 Jan 7;20:11. doi: 10.1186/s12915-021-01219-6 (PMC8742331; doi:10.1186/s12915-021-01219-6)
Supplement: Supplementary file 1 — Additional File 1: Tables S1-S3. Figures S1-S6. Table S1. Genes associated with women’s methylation which correlated with mice expression. Table S2. Primers list (mice). Table S3. Primers list (human). Fig. S1. Weight change in DSS treated and control mice. Fig. S2. Pathway analysis of differentially expressed genes in mice ovaries. Fig. S3. Human Methylation analysis. Fig. S4. Differential SRD5A1 methylation. Fig. S5. Positive controls for steroid treatments in KK-1 cells. Fig. S6. Schematic illustration of the core biological pathways. [file 12915_2021_1219_MOESM1_ESM.docx]

**Epigenetic regulation of 5α reductase-1 underlies adaptive plasticity of reproductive function and pubertal timing**

Ben Bar-Sadeh^1^, Or E. Amichai^1^, Lilach Pnueli^1^, Kurshida Begum^2^, Gregory Leeman^3^, Richard D. Emes^4^, Reinhard Stöger^3^, Gillian R. Bentley^2^, Philippa Melamed^1^

^1^Faculty of Biology, Technion-Israel Institute of Technology, Haifa 32000 ISRAEL

^2^Department of Anthropology, Durham University, Durham, DH1 3LE UK

^3^School of Biological Sciences, University of Nottingham, Nottingham, LE12 5RD UK

^4^School of Veterinary Medicine and Sciences, University of Nottingham, Nottingham, LE12 5RD UK.

Corresponding Author: Philippa Melamed, Faculty of Biology, Technion-Israel Institute of Technology, Haifa 32000 ISRAEL. Tel: 972-4-8293760; [philippa@tx.technion.ac.il](mailto:philippa@tx.technion.ac.il)

**Supplementary Information**

**Table S1: Genes associated with differentially methylated CpGs in the women’s DNA which correlated at one or more CpG with the differential expression of the orthologous gene in the mouse ovaries**

| **gene** | **expression in DSS- treated mice *vs* controls** | **5mC in Bangladeshi women after growing up in Bangladesh rather than the UK** | | | |
| --- | --- | --- | --- | --- | --- |
|  |  | position 1 | position 2 | position 3 | position 4 |
| PKIB | up | down | up |  |  |
| REV3L | up | down |  |  |  |
| CMAH | up | down |  |  |  |
| GIGYF2 | up | down |  |  |  |
| UBR5 | up | down | up |  |  |
| ZBTB38 | up | down | up | up |  |
| BOD1L1 | up | down | down |  |  |
| SCLT1 | up | down | down | down |  |
| FYB | up | down | down | down | down |
| CEP44 | up | down |  |  |  |
| KDM1A | up | up | down |  |  |
| SRD5A1 | down | up | up |  |  |
| SND1 | down | up | up | up |  |

**Table S2: Primers for mice gene and DNA analysis**

| **Gene ID** | **F sequence (5' -> 3')** | **R sequence (5' -> 3')** |
| --- | --- | --- |
| *Rplp0* | GCGACCTGGAAGTCCAACTA | ATCTGCTTGGAGCCCACAT |
| *Srd5a1* | GAATATGTATCTTCAGCCAAC | GGTAATCTTCAAACTTCTCG |
| *Cyp19a1* | CAGTGGAGAGGAGACACTC | CTTCCACCATTCGAACAAGAC |
| *Rasd1* | CTACCATCGAGGACTTCCAC | GACAGGACTTGGTGTCTAGG |
| *Srd5a1* BS outer | AAGGAGTTTTTAGTTAATGTGTGTAG | AAACACAAACTAACACCACCAAAA |
| *Srd5a1 BS* inner | GGAGGTGTTATGTGAAAAATGTTT | CCAAATATCACAAAACTCAACTTC |
| *Gnrh* | GATCCTCAAACTGATGGCCG | CTCCTCGCAGATCCCTGAG |
| *Kiss1* | AGCTGCTGCTTCTCCTCTGT | AGGCTTGCTCTCTGCATACC |
| *Kiss1r* | GGTCATCTACGTTATCTGCC | GCACATGAAGTCTCCCAGC |
| *Esr1* | CTGTGCCGTGTGCAATGACT | CATGCCCACTTCGTAACACT |
| *Tac2* | GTCTCTCTGGAAGGATTGCTG | GGTGTTCTCTTCAACCACGTC |
| *Fkbp5* | GAGTCCAAAGCCTCAGAGTC | GCCAACACCTTCTCGAAGTC |

**Table S3: Primers for human DNA analysis**

| **Island / shore** | **Gene_Name** | **Strand** | **forward primer** | | **reverse primer** |
| --- | --- | --- | --- | --- | --- |
| chr5:6632740-6634162 | *SRD5A1* | Upper | AAGGGTTAGAGTTATTTTGAATGATAG | TTTCATCCCAACAACTCCTTATCCAA | |
|  | *AKAP10* | Lower | TGGTGAAATTTTGGTTAGAGGT | CCAAAATCCTCCAATCTCTTATCAA | |
|  | *PKIB* | Upper | ATGATTTGTTTTTGTTGGTATATAA | CCCTTATACTACATATTTTCAATTCTCTAC | |
| chr7:90893567-90896683 | *FZD1* | Lower | TGGTATTAGAGTGTGAGGGTAAGAG | CCTTTAAACAACTCAACTCCTACAA | |
| chr6:170686006-170687003 | *FAM120B* | Lower | ATTGTTTTGTTTTAAGGGTTTTTTTGTTA | CCTCCCTAATCCCAAATACCTAAA | |
| chr2:26401695-26402099 | *FAM59B* | Upper | GGTTTTGGAGGGGTGGTG | ACCTTCCCCCTCCTAAAAACCTCTA | |
| chr17:43318429-43319243 | *FMNL1* | Upper | AAGGAGTTTGTTGGTGGGTATT | TCCAACTCCTCCACCTTCAAC | |
| chr17:40274523-40275360 | *KAT2A HSPB9* | Upper | GTTGGGGGATTAATTTGTTGTT | AACTACTCTCCTCCCAAACTCC | |
| chr19:15568027-15569227 | *RASAL3* | Upper | TGGGGGTTTTAGGGTATATAAGAGTAG | CAAAACTCCCCTTCCATCTATTACCA | |
| chr1:25255527-25259005 | *RUNX3* | Lower | TTTTAGAGTTTAGGGAGGTGTTT | CTTCCTCTCCCCCCTCCTAAATCTAT | |

**
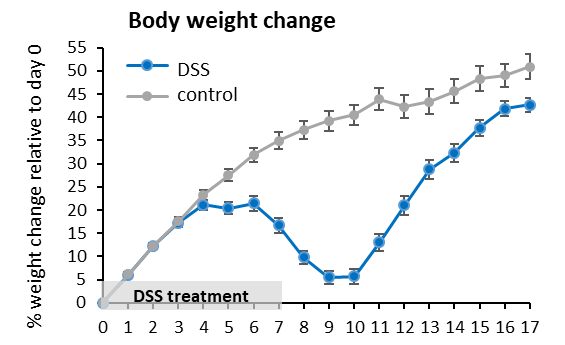
**

**Fig S1**: Changes in body weight in mice after transient p*re-pubertal colitis.* Change in body weight relative to their initial weight are shown for control (n=14-43) and DSS-treated (n=15-48) mice; mean±SEM.

D


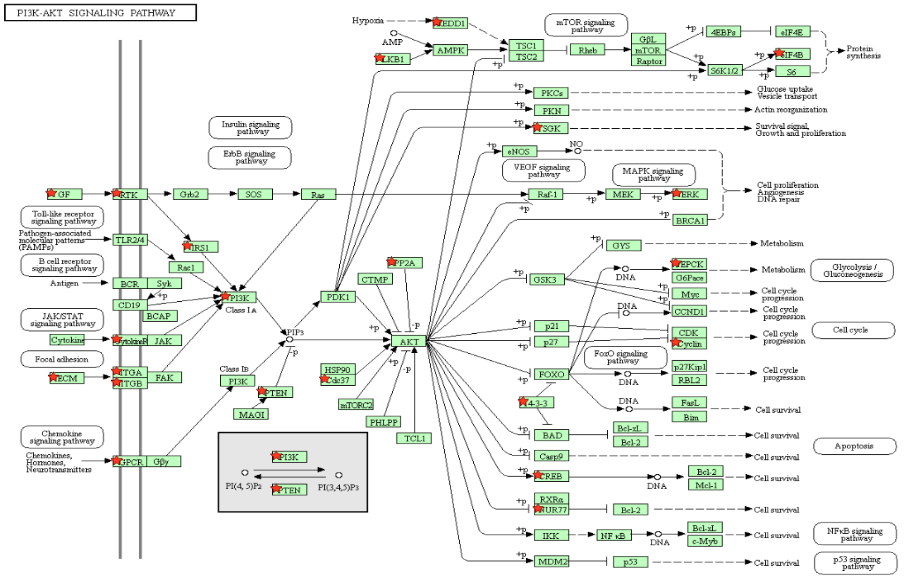

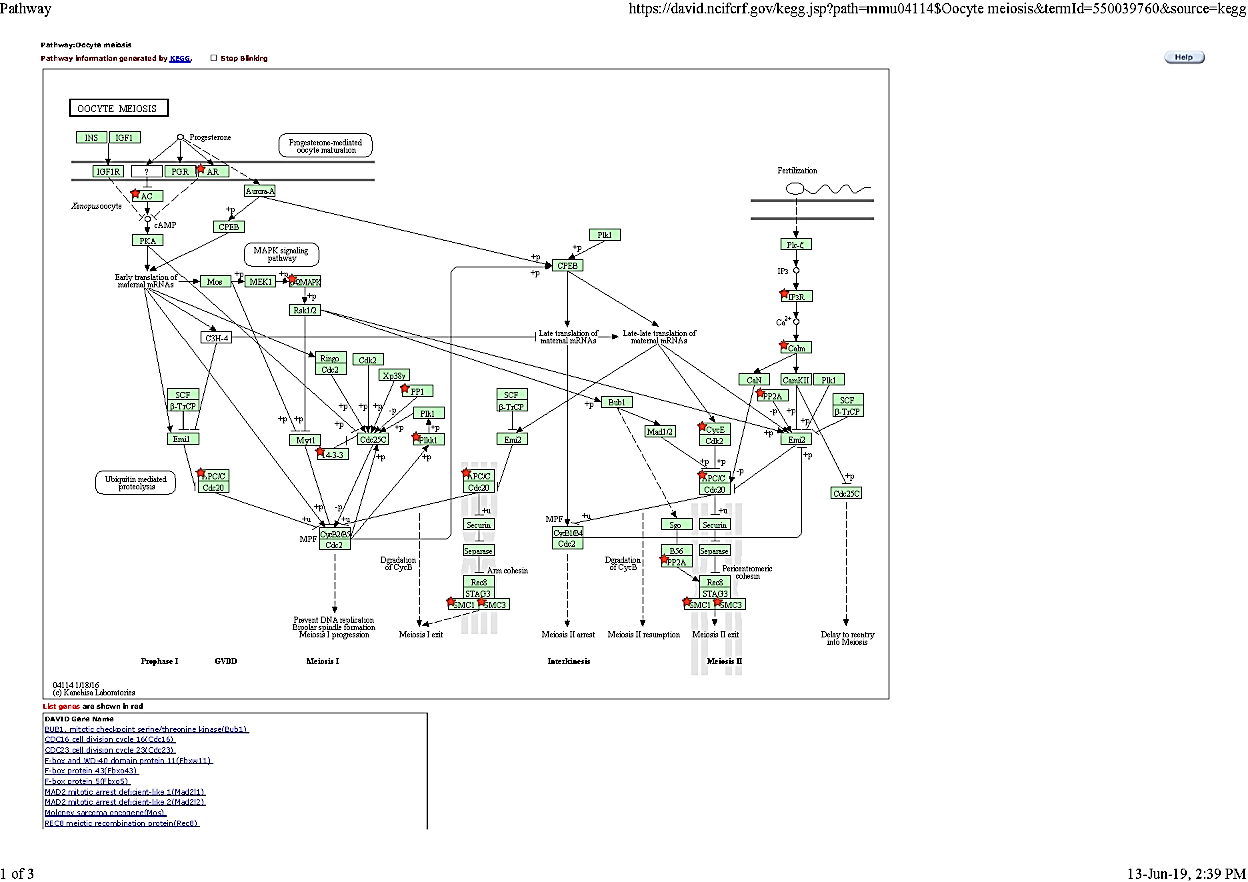

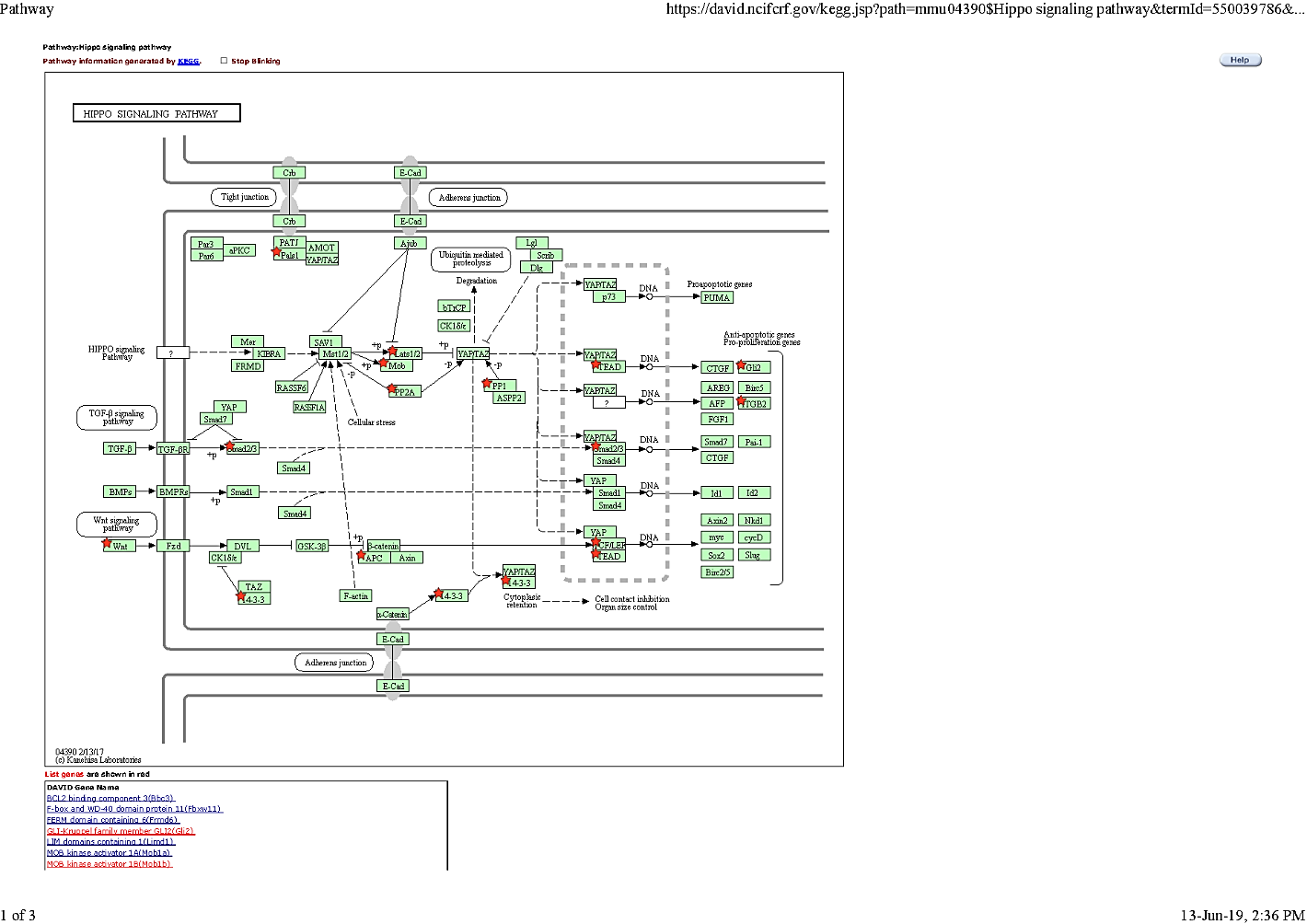

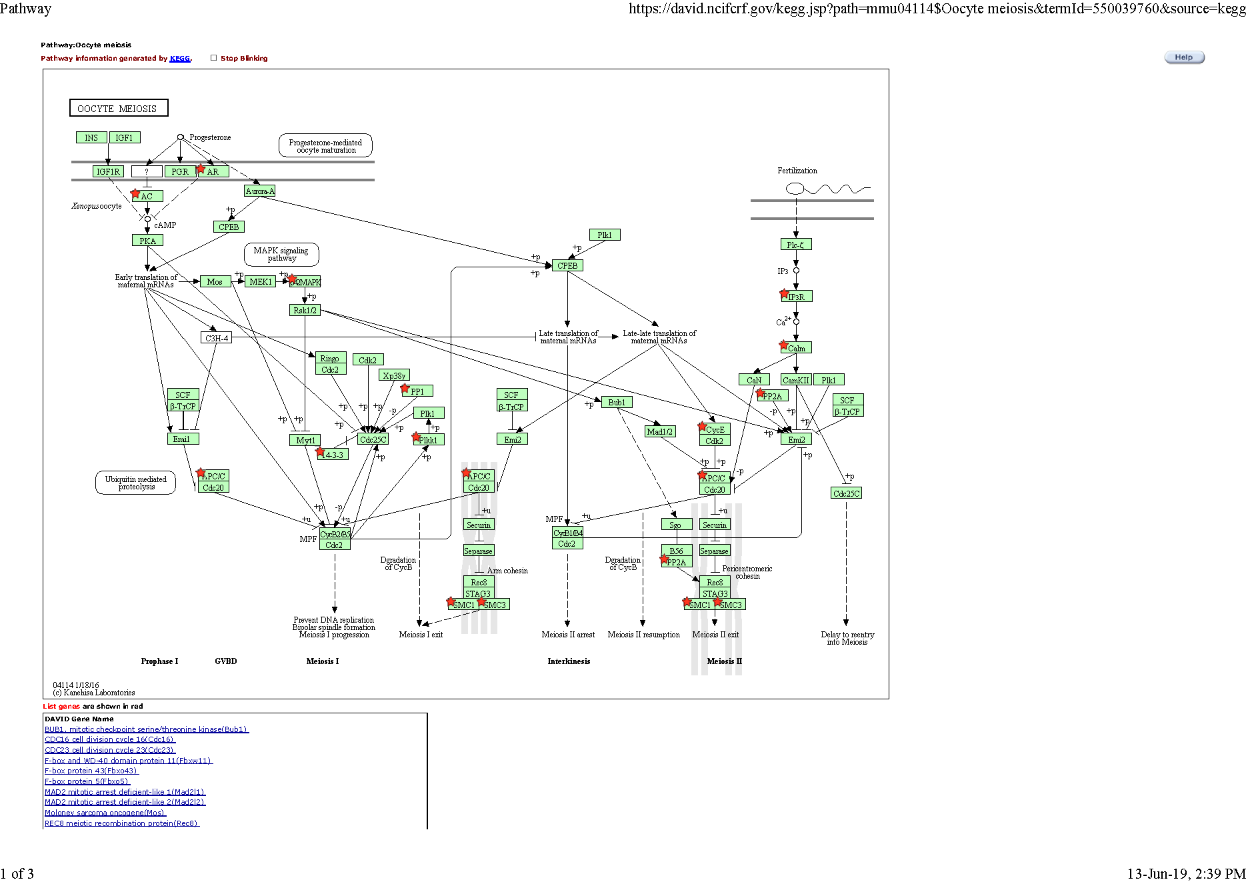

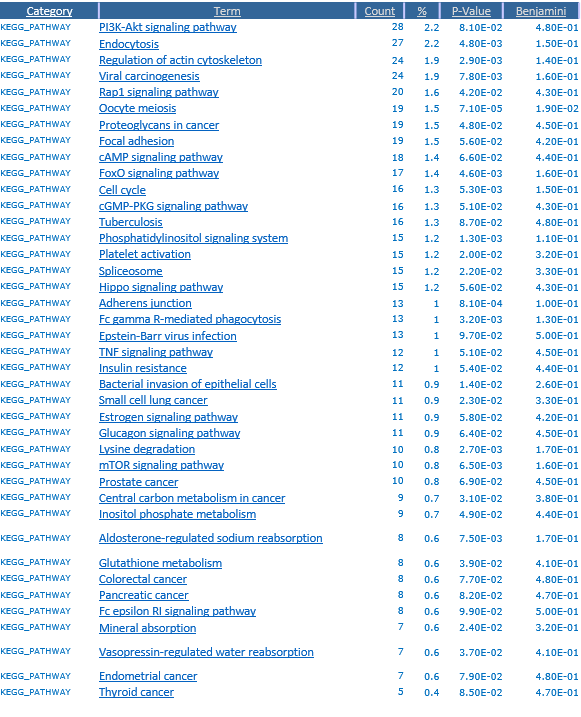


B

C

A

D

**Fig S2**: *Pathway analysis of differentially expressed genes in mice ovaries.* (A) Analysis was performed, using the Database for Annotation, Visualization and Integrated Discovery (DAVID), to determine the enriched pathways in the differentially expressed genes (DEGs) in the DSS-treated *vs* control mice, and the Kyoto Encyclopedia of Genes and Genomes (KEGG) pathways are listed. (B-D) Three of the most enriched pathways (B: PI3K-AKT; C: HIPPO; D: oocyte meiosis) are shown with the DEGs noted by red stars.


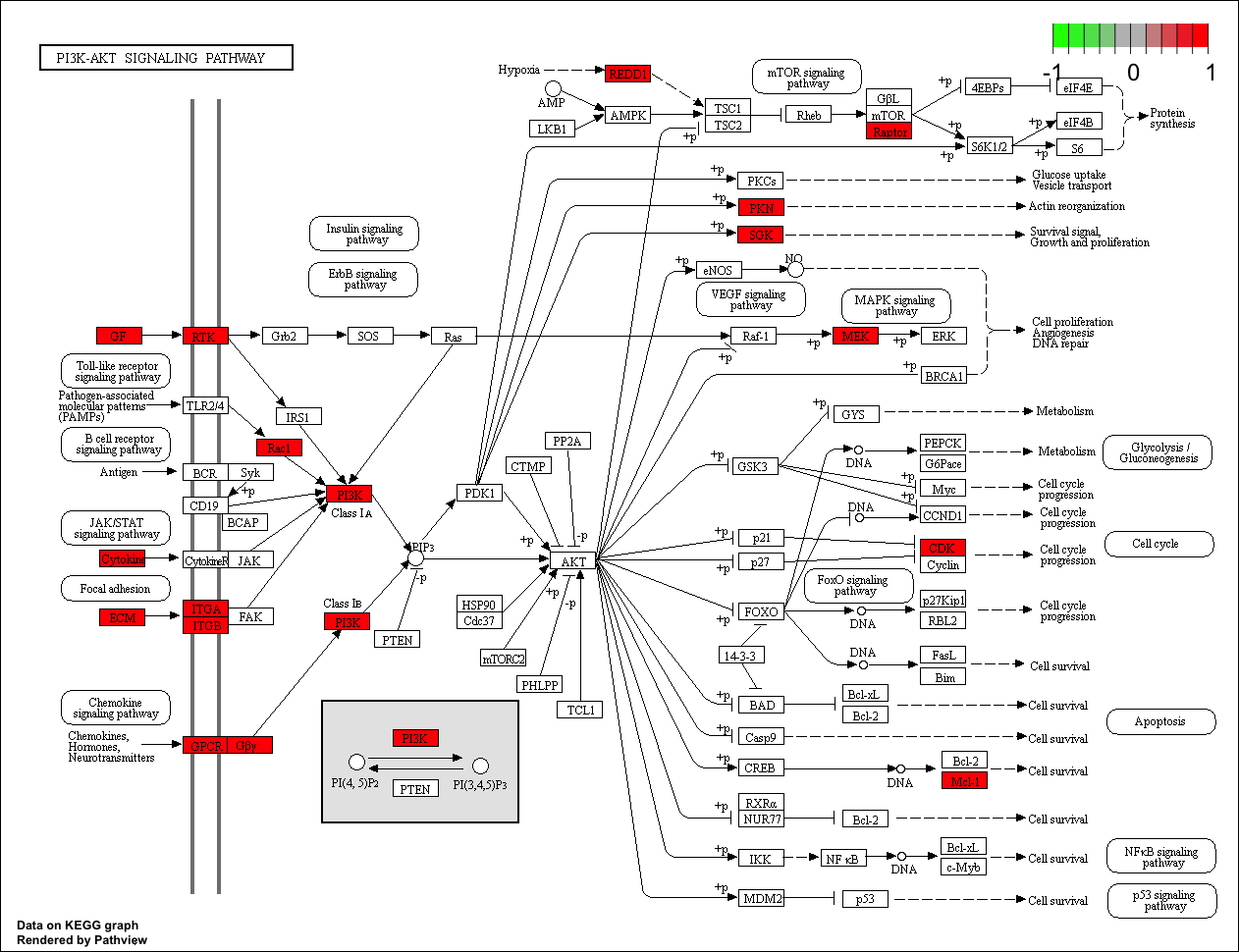

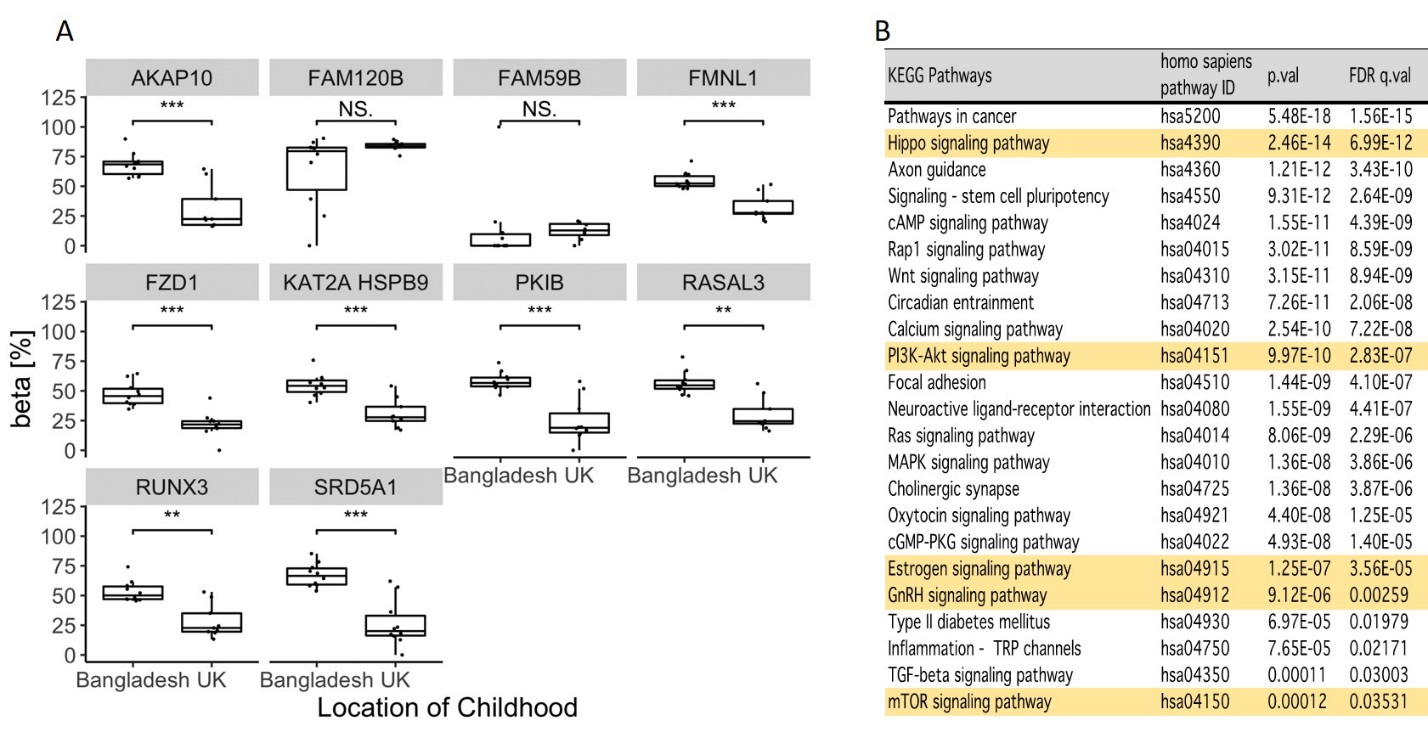
A B C D


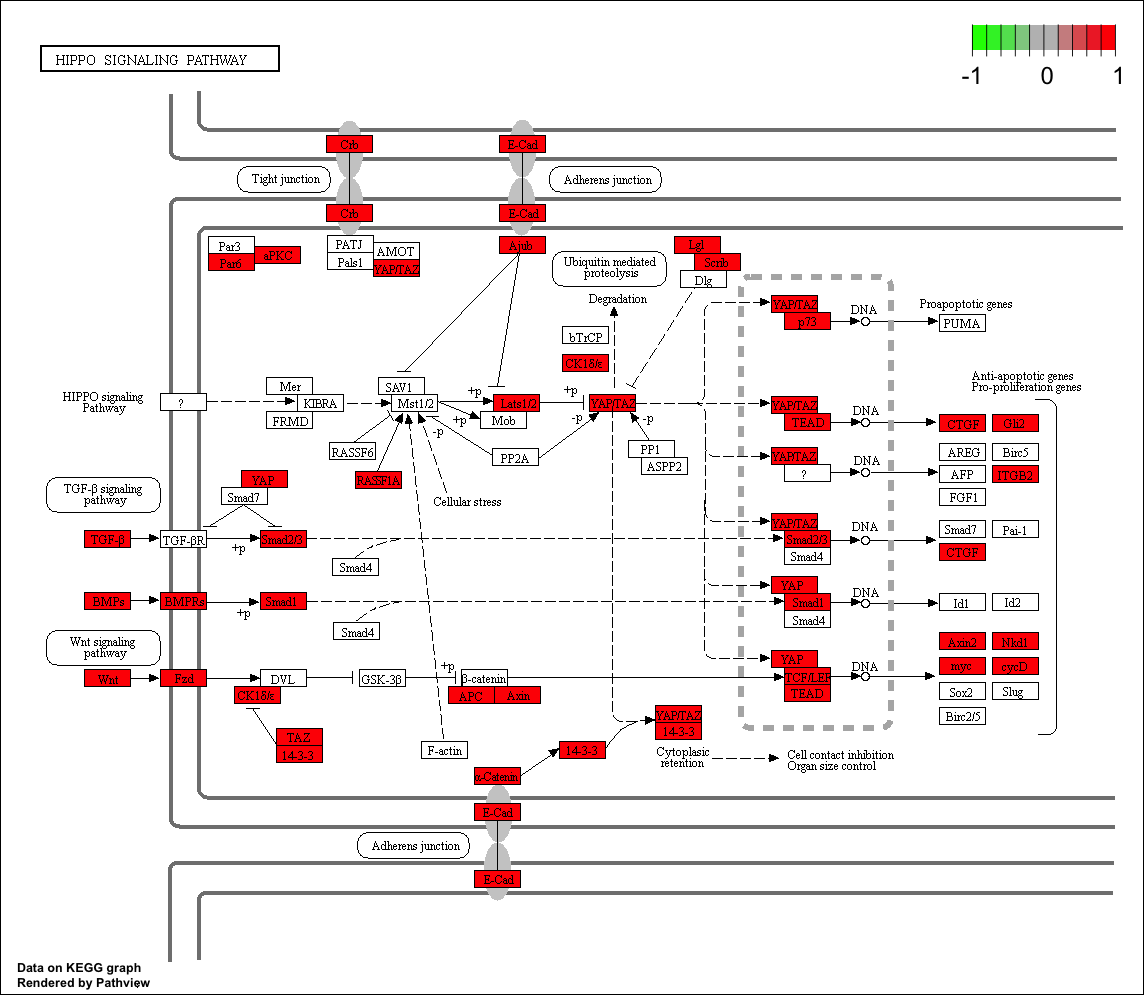

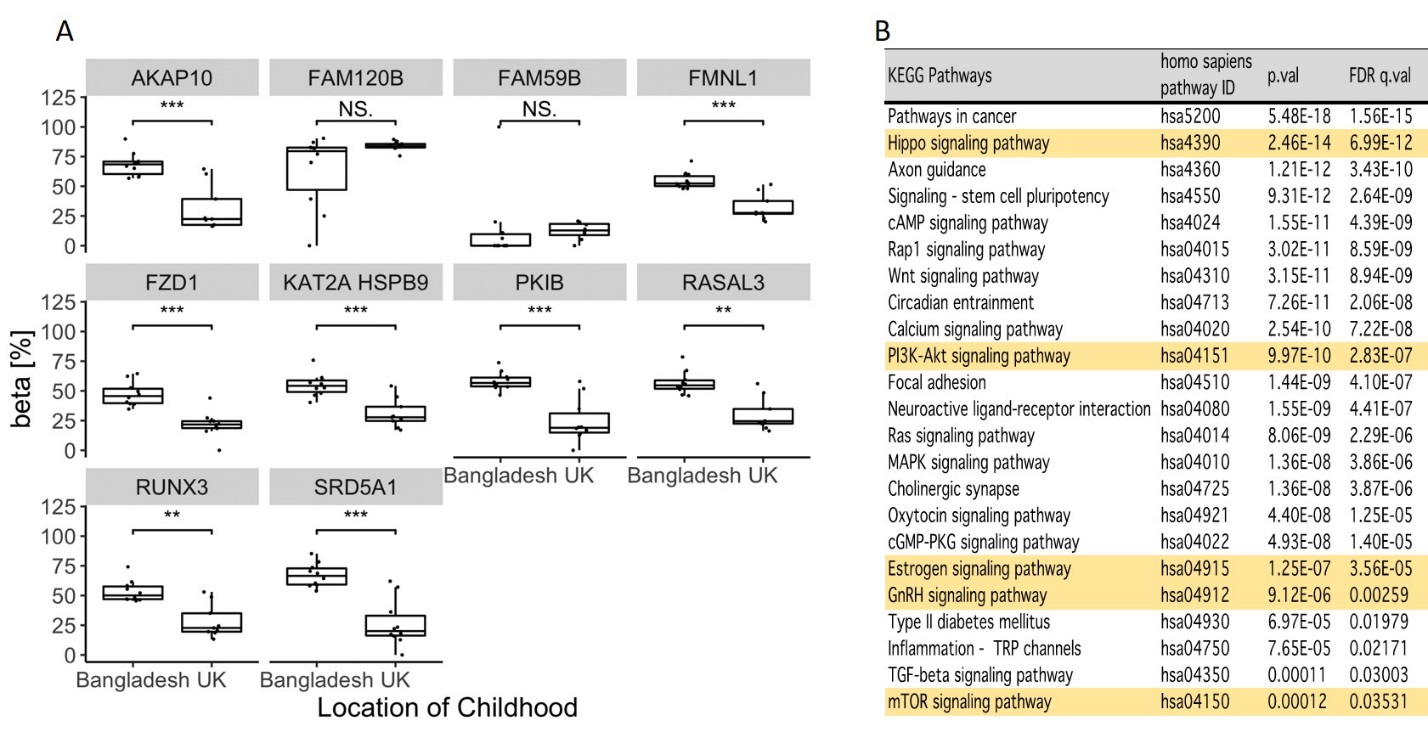


**Fig S3:** *Human Methylation analysis*. (A) KEGG pathway and gene set enrichment analysis of genes associated with differentially methylated probes (FDR <0.05) within islands, shores and shelves. Pathways of particular relevance for our study are highlighted in yellow. (B,C) Genes in the (B) PI3K-Akt (hsa04151) and (C) Hippo (hsa04390) signalling pathways associated with differentially methylated CpGs in islands, shores or shelves are highlighted in red. (D) Targeted bisulfite sequencing confirmation of MethylationEPIC BeadChip data. Beta values: M/(M+U), where M is number of methylated and U un-methylated reads. Bangladesh-childhood (n=10) and UK-childhood (n=9). Mann-Whitney-U test: *** p<0.001; ** p<0.01; * p<0.05; N.S. p>0.05.


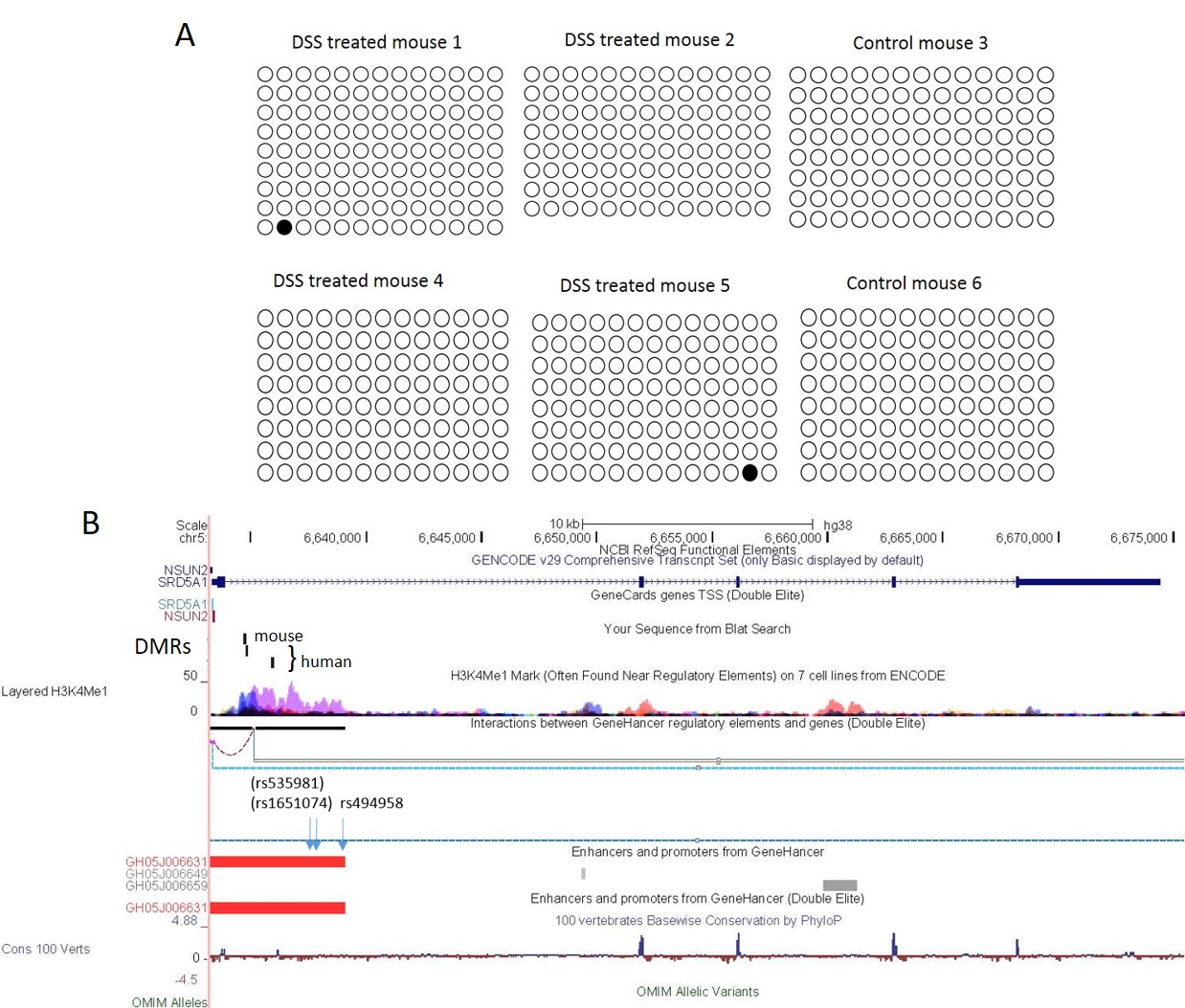


**Fig S4**: *Differential SRD5A1 methylation at a putative enhancer*. (A) After bisulfite conversion of ovarian DNA from DSS-treated (n=4) and control (n=2) mice, the *Srd5a1* proximal promoter was sequenced in multiple clones (rows). Each column represents one CpG (-123 to +62 relative to the TSS); white circles represent unmethylated CpGs and black ones are methylated. (B) The human *SRD5A1* genomic locus in the UCSC genome browser (GRC38/hg38) with observed differentially methylated regions (DMRs) marked, and homologous location of the DMR in the mice ovaries. Also shown are H3K4me1, GENEHancer elements, and an SNP (rs494958) associated with early menopause, and two others in high linkage disequilibrium.


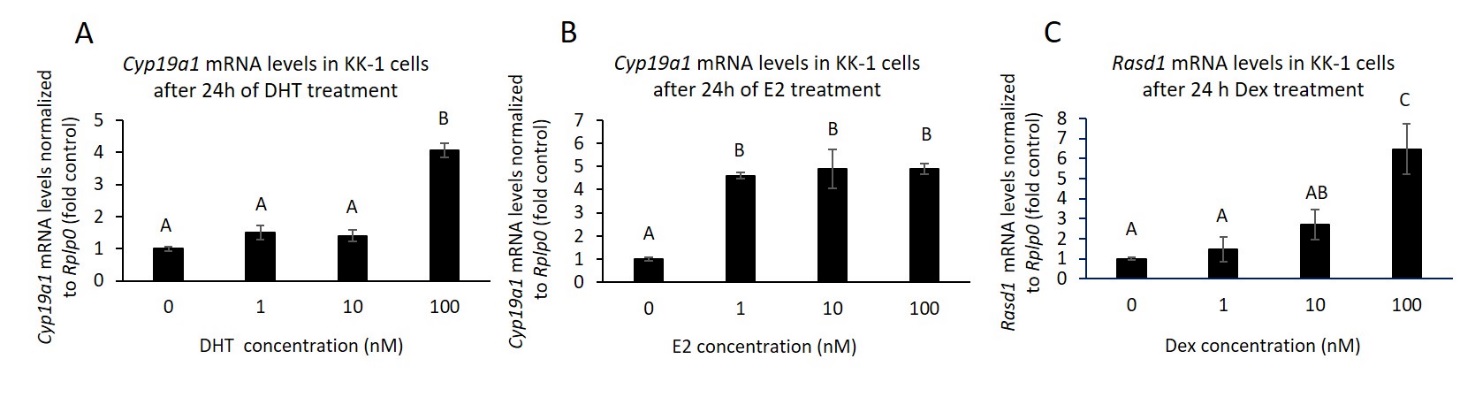


**Fig S5**: *Positive controls for steroid treatments in KK-1 murine ovarian cells.* (A-C) The mRNA from the experiments shown in Fig 4 of the main text was used to measure effects of the treatments on positive control genes (A,B) *Cyp19A1* and (C) *Rasd1*. Mean ± SEM is shown, with ANOVA followed by Tukey-Kramer t-test; means sharing the same letter are statistically similar (P>0.05).


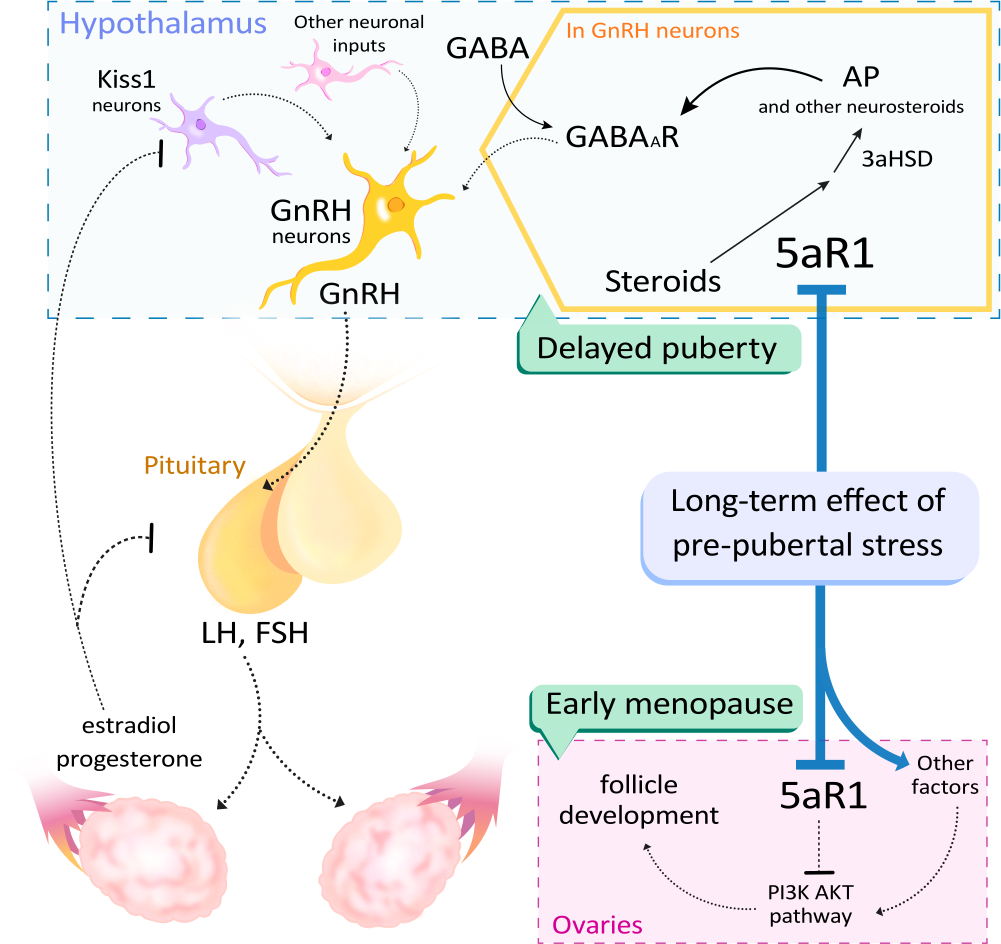


**Fig S6:** *Schematic illustration of the core biological pathways by which early-life stress affects the reproductive axis*. Early-life stress reduces *Srd5a1* expression in the hypothalamus and ovaries. In the hypothalamus, the reduction in 5α reductase-1 (5aR1) lowers neurosteroid levels which could hamper GnRH secretion via GABA_A_R, thereby delaying puberty onset. In the ovaries, 5aRa1 reduction would be expected to increase follicle recruitment by altering the PI3K AKT signaling pathway, resulting in faster depletion of the ovarian reserve and earlier menopause in women. Straight black arrows represent enzymatic reactions, curved arrows represent interaction with GABA_A_R, and dashed arrows or lines represent effects on hormone synthesis or secretion.
